# Tumor vessel sabotage: CD4⁺ T cells enlist macrophages

**DOI:** 10.1038/s41392-026-03019-2

**Published:** 2026-11-05

**Authors:** Stefano Ugel, Francesco De Sanctis

**Affiliations:** Department of Medicine, Section of Immunology, University of Verona, Verona, Italy

**Keywords:** Tumour immunology, Cancer microenvironment, Immunotherapy

In a recent study published in Science, Lian et al. shed light on a previously unrecognized mechanism of CD4 T cell-mediated control of tumor growth that does not require direct tumor cell recognition and can operate independently of interferon (IFN)γ, relying instead on stromal remodeling and destruction of tumor-associated vessels.^1^

Although cancer immunotherapy is often framed around cytotoxic lymphocytes directly recognizing malignant cells, CD4⁺ T cells can also reshape the tumor microenvironment (TME) through cytokine-driven interactions with stromal and immune compartments. In cancer, CD4⁺ T cells promote priming (e.g. dendritic cell licensing) and effector phases of tumor-specific immune responses by locally establishing tertiary lymphoid structures and releasing soluble mediators [e.g., IFNγ and tumor-necrosis factor (TNF)-α], which in turn promote antigen presentation on tumor cells, sustain cytotoxic T cell infiltration, fitness, and tumor killing, as well as shift myeloid cells polarization towards an inflammatory and tumoricidal phenotype. In addition, CD4^+^ T lymphocytes can directly kill tumor cells by recognizing cognate peptide antigens presented by major histocompatibility complex II (MHC II) via T-cell receptor (TCR) engagement. Accordingly, the presence of effector CD4⁺ tumor-infiltrating lymphocytes (TILs) has been associated with favorable outcomes in several settings.^2^ Whether these stroma-remodeling functions can be organized into a therapeutically relevant mechanism of tumor control still represents an unresolved question.

**Fig. 1:**
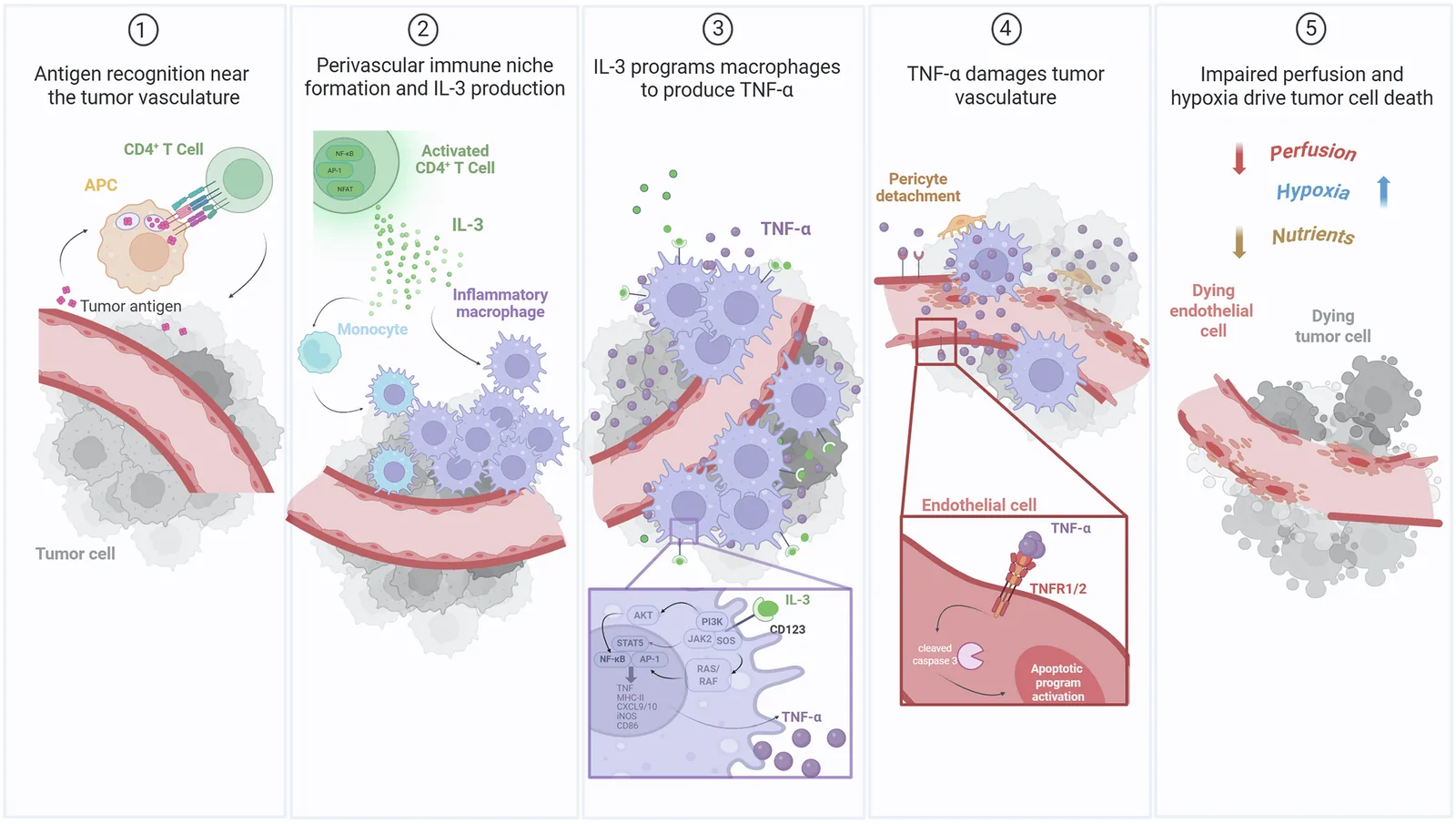
CD4⁺ T cells orchestrate vascular collapse through an IL-3/TNF-α axis. 1. CD4⁺ T cells recognize tumor antigens presented by stromal APCs, rather than by tumor cells, near the tumor vasculature. **2**. Activated CD4⁺ T cells secrete IL-3 and recruit monocytes, which differentiate into inflammatory macrophages, leading to the formation of a perivascular immune niche. **3**. IL-3 signals through IL-3Rα (CD123) on macrophages, activating JAK/STAT, PI3K/AKT, and MAPK signaling and inducing TNF-α production. **4**. TNF-α activates an NF-kB-dependent cell-death program in vascular stromal cells, driving pericyte detachment, endothelial apoptosis, vascular leakage, and loss of vessel integrity. **5**. Vascular collapse results in reduced perfusion, nutrient deprivation, hypoxia, and, lastly, extensive tumor cell death. APC Antigen presenting cell, AKT Protein Kinase B, AP-1 Activator Protein 1, NFAT Nuclear Factor of Activated T-cells, JAK Janus Kinase, NF-kB Nuclear Factor kappa-light-chain-enhancer of activated B cells, iNOS inducible nitric oxide synthase, PI3K Phosphoinositide 3-kinase, RAF Rapidly Accelerated Fibrosarcoma, RAS rat sarcoma SOS Son of sevenless, STAT Signal Transducers and Activators of Transcription. Figure created with Biorender

Lian and colleagues employed different transplantable solid tumor models (colon, breast, and melanoma) engineered to express MHC-II-restricted antigens recognized by tumor-specific CD4⁺ T cells (LCMV glycoprotein [GP] containing the gp66 MHC-II epitope, TRP1 endogenous melanoma antigen), and several transgenic hosts to mechanistically unravel a novel CD4-dependent tumor-restriction pathway. The authors demonstrated that GP66-specific CD4⁺ T cells (Smarta), adoptively transferred into immunodeficient (Rag1^−/−^ or Rag2^−/−^) tumor-bearing mice, controlled the growth of MHCII-proficient (MC38) and MHCII-deficient (C10 clone derived from MC38) GP66-expressing cell lines. Integrating multiple immunofluorescence with spatial transcriptomics and sc-RNAseq of tumor-infiltrating immune subsets, the authors revealed a treatment-dependent establishment of perivascular immune clusters organized around endothelial structures and enriched in CD4^+^ T lymphocytes and tumor-associated macrophages (TAMs) with inflammatory (*Il1b, Nos2, Cxcl9*) and antigen presentation (*Cd74, Cd86, Ciita*) features (Fig. 1). Accordingly, perivascular immune organization and therapeutic efficacy could be dampened in *Ccr2*^*−/−*^ *Cx3cr1*^*−/−*^ hosts. A similar molecular analysis of TILs identified eight clusters of antigen-activated T cells, including resident, non-cycling cells expressing IFNγ, interleukin (IL)- 3, granulocyte-macrophage colony-stimulating factor (GM-CSF), and TNF-α. The authors untangled the role of CD4-released cytokines in promoting tumor control, employing both genetic (Ifng^−/−^ vs Ifng^−/−^ Tnf^−/−^ vs Ifng^−/−^ Il3^−/−^ Smarta T cells) and pharmacological targeting approaches (anti-IL-3, GM-CSF, TNF-α). Although blockade of IL-3, GM-CSF, or TNF-α impaired tumor control, CRISPR-Cas9 genetic inactivation of Tnf, but not Il3, in Smarta cells had no detectable effect (IFNγ provided a dismal additive effect). Thus, among the tested cytokines, CD4-derived IL-3 was indispensable for mediating treatment efficacy.

IL-3, historically known for promoting hematopoiesis and the differentiation of T lymphocytes, has recently been recognized as a pleiotropic cytokine involved in many inflammatory diseases, given its ability to sustain leukocyte survival, proliferation, polarization, and recruitment. T lymphocyte-released IL-3 triggers a self-perpetuating mono macrophage-T cell recruitment and network axis through the release of CCL2 CCL7 and CCL12 chemokines in central nervous system autoimmunity^3^ and amplifies emergency myelopoiesis and inflammatory cytokine production in sepsis.^4^ Given the pro-angiogenic and proliferative actions of IL-3 on endothelial and epithelial cells, and the few reports linking IL-3 to immune checkpoint efficacy, its role in cancer remains debated and likely context-dependent. Lian and colleagues extended these observations to the cancer immunotherapy field and identified macrophages as the critical IL-3-responsive population in tumor immunity. Smarta-derived IL-3 triggered local TNF-α release from inflammatory TAMs, likely via IL-3Rα/CD123-dependent activation of the JAK/STAT, PI3K/AKT, and MAPK pathways (Fig. 1). Macrophage-derived TNF-α acted not on tumor cells, whose TNFR1/2 expression was dispensable, but on vascular stromal cells, promoting pericyte loss, endothelial cell apoptosis, vessel widening, and erythrocyte extravasation. Vascular damage impaired perfusion, reduced oxygen and nutrient supply, increased hypoxia, and culminated in tumor cell death (Fig. 1). This bystander tumor growth restriction relies on T cells’ ability to synergize with TAMs to target non-immune stromal subsets, demonstrating vascular disruption as an adaptive immune mechanism of tumor control. This “smart” strategy to indirectly tackle tumor growth by targeting tumor endothelium has several advantages: first, it does not require TCR-MHCII interaction between lymphocytes and tumor cells (potentially less vulnerable to tumor-cell MHC-II loss or antigen-presentation defects); second, it is a self-amplifying mechanism that relies on TAM support, which are abundantly present in tumors. Third, tumor-associated vascular cells are genetically more stable than malignant cells, and their damage can generate broad bystander effects across the TME. Fourth, tumor-associated vascular-targeting strategies can promote antigen spreading and expansion of T cell responses to secondary epitopes, as shown preclinically.^5^

Immune-dependent vascular targeting is conceptually distinct from vascular normalizing treatments. Anti-angiogenic therapies relieve hypoxia, increase perfusion, immune cell trafficking, and the efficacy of immune checkpoint inhibitor (ICI) therapy. Lian et al. describe a late effector stage of adaptive immunity targeting tumor vasculature and resulting in vessel collapse, increased hypoxia, and tumor damage. This latter approach can provide substantial differences in immune cell infiltration, whereas heightened hypoxia may activate tumor immune-evasive routes, including angiogenesis, immunosuppression, and metabolic adaptation, all leading to therapy resistance. These considerations suggest potential synergy and temporal sequencing between the two approaches.

From a clinical perspective, Lian and colleagues identified similar spatial organization of CD4^+^ T lymphocytes, TAMs, and vascular cells in human ovarian tumors, where inflammatory macrophage signatures associated with improved survival and nearby tumor cells displayed hypoxia and necrosis. These findings raise several questions. Can perivascular immune hubs be identified by routine histological analysis as marker of ongoing tumor-specific immunity? Do they predict response to ICI? In which tumor type and treatment settings do they arise? Although most of the evidence still derives from preclinical models, engineered tumors in an “adoptive cell therapy” setting, and patient-level validation across different cancer treatments remain essential, this study broadens the logic of cancer immunotherapy and of CD4^+^ T cell functions, from helper to saboteur of the supply lines.

This discovery opens exciting opportunities to develop stromal-directed immunotherapies that prevent antigen loss and immune escape, new combination approaches, and proposes immune-mediated vascular disruption as an important complement to existing cytotoxic immunotherapies.
